# Misfolded SOD1 pathology in sporadic Amyotrophic Lateral Sclerosis

**DOI:** 10.1038/s41598-018-31773-z

**Published:** 2018-09-21

**Authors:** Bastien Paré, Manuela Lehmann, Marie Beaudin, Ulrika Nordström, Stephan Saikali, Jean-Pierre Julien, Jonathan D. Gilthorpe, Stefan L. Marklund, Neil R. Cashman, Peter M. Andersen, Karin Forsberg, Nicolas Dupré, Peter Gould, Thomas Brännström, François Gros-Louis

**Affiliations:** 1 Laval University Experimental Organogenesis Research Center/LOEX, Division of Regenerative Medicine, CHU de Québec Research Center – Enfant-Jésus Hospital, Québec, Canada; 2 Department of Surgery, Faculty of Medicine, Laval University, Québec, Canada; 3 Department of Pharmacology and Clinical Neuroscience, Umeå University, Umeå, Sweden; 4 Neuroscience Division of the CHU de Québec and Department of Medicine of the Faculty of Medicine, Laval University, Québec, QC Canada; 5 Department of Medical Biology, Division of Anatomic Pathology and Neuropathology, CHU de Québec, Hôpital de l’Enfant-Jésus, Québec, Canada; 6 Department of Psychiatry and Neuroscience, Laval University, Québec City, Québec, Canada; 7 Centre de Recherche CERVO, Québec City, Québec, Canada; 8 Department of Medical Biosciences, Clinical Chemistry, Umeå University, Umeå, Sweden; 9 Department of Medicine (Neurology), Brain Research Center, University of British Columbia, Vancouver, BC Canada; 10 Department of Medical Biosciences, Pathology, Umeå University, Umeå, Sweden

## Abstract

Aggregation of mutant superoxide dismutase 1 (SOD1) is a pathological hallmark of a subset of familial ALS patients. However, the possible role of misfolded wild type SOD1 in human ALS is highly debated. To ascertain whether or not misfolded SOD1 is a common pathological feature in non-*SOD1* ALS, we performed a blinded histological and biochemical analysis of *post mortem* brain and spinal cord tissues from 19 sporadic ALS, compared with a *SOD1* A4V patient as well as Alzheimer’s disease (AD) and non-neurological controls. Multiple conformation- or misfolded-specific antibodies for human SOD1 were compared. These were generated independently by different research groups and were compared using standardized conditions. Five different misSOD1 staining patterns were found consistently in tissue sections from SALS cases and the SOD1 A4V patient, but were essentially absent in AD and non-neurological controls. We have established clear experimental protocols and provide specific guidelines for working, with conformational/misfolded SOD1-specific antibodies. Adherence to these guidelines will aid in the comparison of the results of future studies and better interpretation of staining patterns. This blinded, standardized and unbiased approach provides further support for a possible pathological role of misSOD1 in SALS.

## Introduction

Amyotrophic Lateral Sclerosis (ALS) is a heterogeneous neurodegenerative syndrome characterized by adult-onset progressive loss of primarily motor neurons in the cerebral cortex, brain stem, and spinal cord. ALS leads to progressive muscle weakness, atrophy and typically death 3 to 5 years after symptom onset^1,2^. About 5–10% of ALS cases are hereditary (familial; FALS)^3,4^. The remaining ≈90% of cases lack an overt familial history and are referred to as sporadic (SALS)^3–5^. Repeat DNA expansion in *C9ORF72* as well as mutations in approximately 40 other genes have been associated with ALS; most frequently *SOD1*, *TARDBP* (TDP-43) and *FUS/TLS* (for review see^6^).

Neurodegeneration in ALS is associated with protein misfolding and aggregation and the formation of inclusions in regions of the central nervous system (CNS) affected by the disease^7^. For example, cytoplasmic inclusions of TDP-43 are frequently observed in motor neurons in a majority of non-*SOD1* FALS cases, with or without *TARDBP* mutations, and in many SALS cases^8–17^. Inclusions of human mutant or wild type SOD1 are found in transgenic mouse models overexpressing the proteins^18–24^ and in patient material at autopsy^8,19,25–28^. The findings that SOD1 mutant proteins with biophysical properties similar to wild type SOD1 (wtSOD1); including D90A^29^ and L117V^30^, cause ALS, as well that overexpression of wtSOD1 causes an ALS-like disease in transgenic mice^24^, supports an emerging hypothesis that wtSOD1 contributes to ALS. Interestingly, it has been also reported that wtSOD1 can acquire an aberrant conformation, implying a possible shared pathological pathway between mutant *SOD1*-linked FALS and SALS^27^. Other studies have also demonstrated that wild type human SOD1 acquires toxic properties upon oxidative damage^31–33^. It also lead to an exacerbation of disease phenotype in transgenic mice expressing different SOD1 mutants^34,35^. Hence, it is possible that wtSOD1 may be a contributor to disease pathogenesis in sporadic ALS.

Conformational- and misfolded-specific antibodies, recognizing either naturally buried epitopes or completely misfolded forms of SOD1 have emerged as valuable tools with which to distinguish unfolded or misfolded SOD1 species from the natively folded protein^20,26–28,36,37^. These antibodies have been used to detect misSOD1 in spinal motor neurons of SALS patients without *SOD1* mutations^19,26–28^. However, several other studies have concluded that misSOD1 is not present in SALS^38–42^. Since this discrepancy could depend on differences in the methodology used for immunohistochemistry, we aimed to address this in a range of SALS patient material. Using a panel of different antibodies that bind specifically to unfolded/misfolded conformations of SOD1 and following standardized experimental protocols, we have undertaken a blinded histopathological study and also biochemical analysis of misSOD1. The results provide supporting evidence that strongly suggest a role for misSOD1 in non-*SOD1* SALS.

## Results

### Specific staining of misSOD1 in FALS

**Figure 1:**
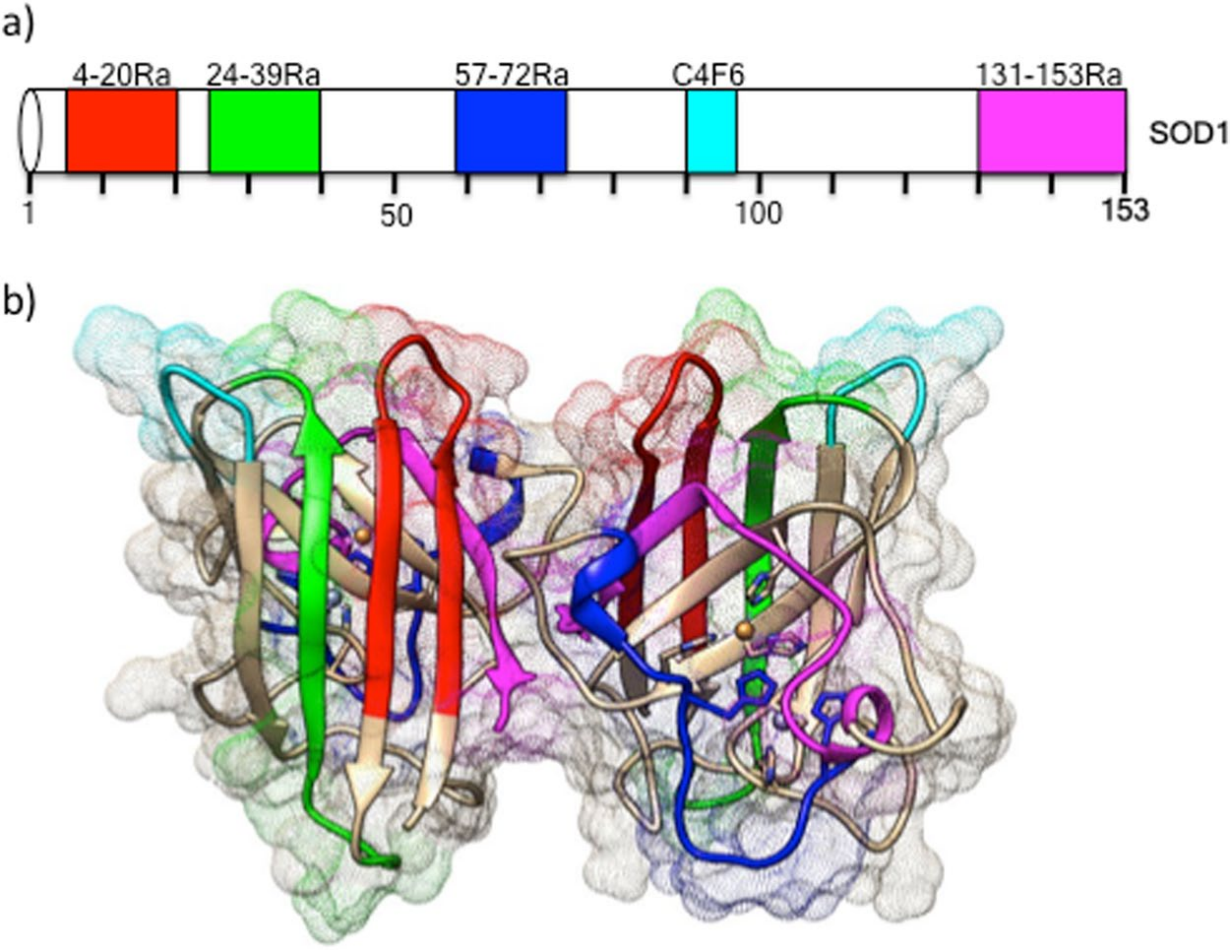
Human SOD1 protein schematic representation with highlighted misSOD1 epitope mapping regions (**a**) Linear representation of the 5 tested misfolded/conformational SOD1 specific antibody’s binding regions. (**b**) 3D protein structure representation with highlighted misfolded/conformational SOD1 antibody’s bindging regions. Ra: Rabbit polyclonal. C4F6: mouse monoclonal.

**Figure 2:**
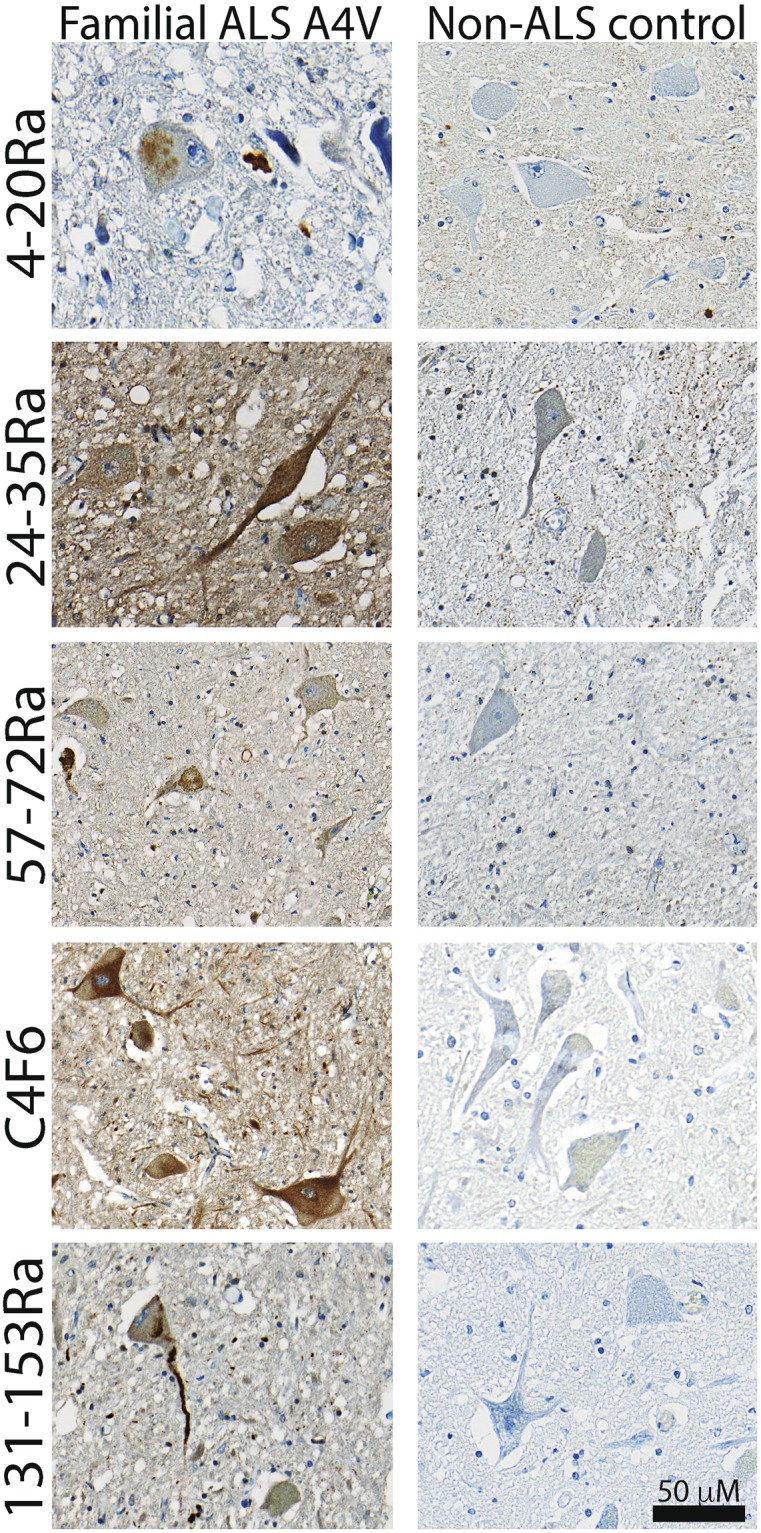
Immunodetection of misSOD1 accumulation in spinal cord of A4V SOD1 FALS patient Lumbar spinal cord sections from A4V-SOD1 FALS autopsied patient (positive control) and non-neurological control individual (negative control) immunostained with 5 misSOD1 conformational antibodies (4–20Ra, 24–39Ra, 57–72Ra, C4F6 and 131–153 R). Characteristic misSOD1-positive cytoplasmic accumulation in spinal motor neurons can be observed with all tested antibodies in the A4V-SOD1 FALS patient, whereas no immunostaining is detected in the non-neurological control. Scale bar: 50 mm.

Four different polyclonal, as well as one monoclonal antibody, covering epitopes across the entire SOD1 protein (N-terminus, central region and C-terminus) were used (Fig. 1). All antibodies chosen have previously been shown to be highly specific for human misSOD1^26–28^. First, immunohistochemical analyses were performed in post-mortem spinal cord tissues from a FALS patient heterozygous for the A4V *SOD1* mutation (positive control; Fig. 2) and compared with 2 non-neurological controls (negative controls), in order to standardize the immunostaining protocol.

All 5 antibodies revealed the presence of cytoplasmic misSOD1 accumulation within spinal motor neurons of the grey matter in the FALS patient harbouring the A4V *SOD1* mutation. Strong axonal and dendritic misSOD1 accumulation, as well as intense punctate immunostaining in the neuropil were also observed. In agreement with previous observations^26,28^, no immunoreactive signal was detected in the control spinal cord sections, confirming the high specificity of all of the tested misSOD1 antibodies.

### Widespread staining of misSOD1 in SALS

**Figure 3:**
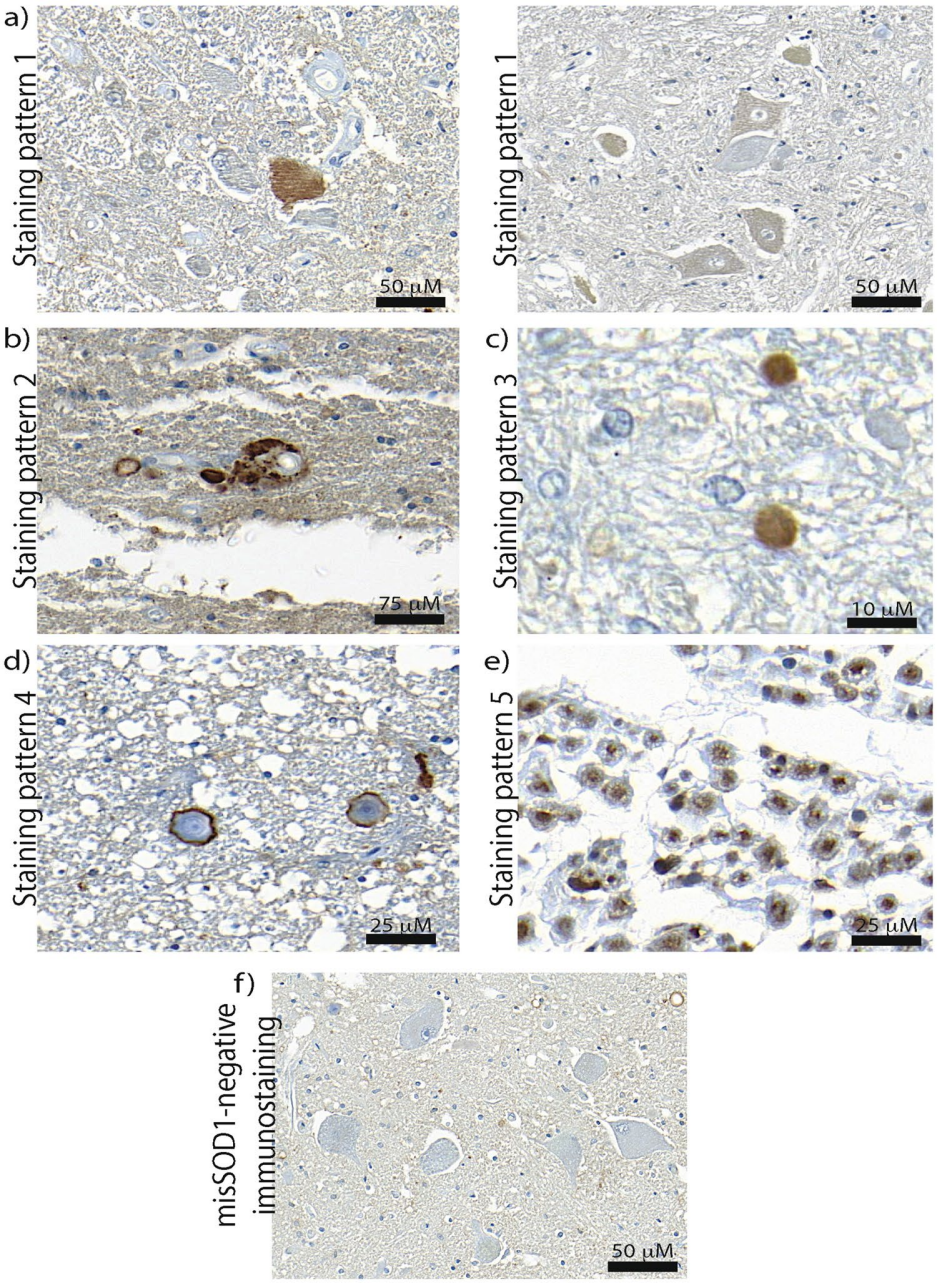
Detected misSOD1 immunostaining patterns MisSOD1 immunostaining patterns detected in SALS individuals were (**a**) Diffuse misSOD1 staining in the cytoplasm of motor neurons (immunostaining pattern 1). Note that different misSOD1 immunosignal intensity levels were detected. As misSOD1-positive motor neurons can be detected beside misSOD1-negative motor neurons on the same tissue section, it is unlikely that the observed misSOD1 accumulation represents a false-positive signal (**b**) misSOD1-positive deposits detecetd in the cytoplasm of motor neurons (immunostaining pattern 2) (**c**) Dendritic and axonal misSOD1 accumulation (immunostaining pattern 3) (**d**) MisSOD1-positive perivacuolar ring-like structures (immunostaining pattern 4) (**e**) MisSOD1 nuclear accumulation (immunostaining pattern 5) and (**f**) misSDO1-negative immunostaining observed in non-neurological control. Scale bars are presented in the lower right corner for each detected immunostaining patterns.

Having validated the panel of SOD1 antibodies in the FALS vs control material, we next analysed a cohort of 13 French Canadian SALS cases, 2 neurological AD cases and 2 non-neurological controls (Table 1). Material from cortical (frontal, temporal and occipital lobes as well as entorhinal sections) and spinal cord (lumbar, cervical, thoracic and sacral spinal sections) regions were examined in a blinded manner. Positive misSOD1 immunoreactive signals were detected in all of the 13 SALS cases using the 5 misSOD1 antibodies (Fig. 3, Supplementary Table 1 and Supplementary Figures 1–5). Five characteristic misSOD1-positive immunostaining patterns were observed and can be summarized as; (1) diffuse misSOD1 staining in the cytoplasm of motor neurons, (2) misSOD1-positive deposits in the cytoplasm of motor neurons, (3) dendritic and axonal misSOD1 accumulation, (4) misSOD1-positive perivacuolar ring-like structures, and (5) misSOD1 nuclear accumulation (Fig. 3). We determined a cut-off, whereby to be considered as misSOD1positive, a case should display more than one of the above immunostaining patterns in two or more regions of the CNS. Interestingly, not all tested antibodies detected the misSOD1 immunostaining patterns with the same intensity in each of the patients (Supplementary Figures 1–5). Based on these results, more than one conformational- and misSOD1-specific antibody was consequently used to detect the presence of misSOD1 species in at least two different spinal cord regions for each of the tested SALS and control individuals.

**Table 1:** Summary of the studied ALS patients and control individuals.

Patient	Genotype	Age of onset	Age of death	Time to autopsy (Hours)	Gender	Pathological TDP-43 status	Origin
1	FALS SOD1/A4V	72	73	22	F	Nuclear	Umeå, Sweden
2	SALS	53	63	16	F	Cytoplasmic	Québec, Canada
3	SALS	68	78	18	M	Cytoplasmic	Québec, Canada
4	SALS	53	63	15	F	Cytoplasmic	Québec, Canada
5	SALS	79	81	22	M	Cytoplasmic	Québec, Canada
6	SALS	52	54	Unavailable	M	Cytoplasmic	Québec, Canada
7	SALS	Unavailable	73	Unavailable	M	Cytoplasmic	Québec, Canada
8	SALS	60	67	Unavailable	F	Cytoplasmic	Québec, Canada
9	SALS	72	74	Unavailable	M	Cytoplasmic	Québec, Canada
10	SALS	72	74	23	M	Cytoplasmic	Québec, Canada
11	SALS	49	53	20	F	Cytoplasmic	Québec, Canada
12	SALS	Unavailable	61	15	M	Cytoplasmic	Québec, Canada
13	SALS	Unavailable	78	24	F	Cytoplasmic	Québec, Canada
14	SALS	67	69	24	M	Cytoplasmic	Québec, Canada
15	SALS	68	70	21	M	Cytoplasmic	Umeå, Sweden
16	SALS	60	61	23	F	Cytoplasmic	Umeå, Sweden
17	SALS	64	65	18	M	Cytoplasmic	Umeå, Sweden
18	SALS	62	64	20	M	Cytoplasmic	Umeå, Sweden
19	SALS	64	69	Unavailable	F	Cytoplasmic	Umeå, Sweden
20	SALS	64	67	Unavailable	F	Cytoplasmic	Umeå, Sweden
21	Non-neurological Control	Not applicable	65	15	M	Nuclear	NIH Neurobiobank, USA
22	Non-neurological Control	Not applicable	73	22	M	Nuclear	Umeå, Sweden
23	Neurological Control (Alzheimer)	Not applicable	65	23	F	Nuclear	Québec, Canada
24	Neurological control (Alzheimer)	Not applicable	58	20	M	Nuclear	Québec, Canada

**Table 2:** Immunohistochemistry conditions using the Ventana BenchMark ULTRA.

Antibody	Antigen retrieval incubation time	Working dilution
4–20Ra	44 minutes	1/350
24–39Ra	44 minutes	1/1,000
57–72Ra	24 minutes	1/3,000
C4F6 (Non-purified)	44 minutes	1/2
131–153Ra	44 minutes	1/1,000

**Table 3:** Studies describing the presence or absence of misSOD1 in human SALS post-mortem tissues.

	misSOD1	# of SALS cases
Bosco *et al*., 2010, *Nat. Neuro*^27^.	Yes	9
Forsberg *et al*., 2010, *PLoS ONE*^26^	Yes	29
Forsberg *et al*., 2011, *Acta Neuropatho*^28^.	Yes	51
Pokrishevsky *et al*., 2012, *PLoS ONE*^8^	Yes	3
Grad *et al*., 2014, *PNAS*^19^	Yes	20
Ayers *et al*., 2014, *Acta Neuropatho. Comm*^41^.	Yes but no difference with controls	25
Brotherton *et al*., 2012, *PNAS*^39^	No	25
Liu *et al*., 2009, *Ann. Neuro*^38^.	No	13
Kerman *et al*., 2010, *Acta Neuropatho*^40^.	No	10
Da Cruz *et al*., 2017, *Acta Neuropatho*^42^.	Yes but no difference with controls by IHC and no misSOD1 detection in SALS by IF	30

**Table 4:** Summary of the proposed guidelines.

	Guidelines
1	To use more than one misfolded SOD1/conformational-specific antibody before concluding any results
2	To test and use optimal antibodies working concentrations
3	To optimize antigen retrieval time for each antibody
4	To use citrate-based instead of TRIS/EDTA-based buffers
5	To test different central nervous system regions (cervical, lumbar, thoracic spinal cord sections and other brain regions)
6	To perform, in parallel, Hematoxylin/Eosin coloration on adjacent sections

### Distinct patterns of misSOD1 in SALS

Immunohistopathological analyses using the 4–20Ra misSOD1 antibody, targeting the N-terminal region of the human SOD1 protein, showed diffuse cytoplasmic misSOD1 accumulation in spinal motor neurons of all SALS cases (Supplementary Figure 1, and Supplementary Table 1). Axonal segments in the ventral horn, perivacuolar ring-like structure, as well as nuclear misSOD1 accumulation were identified SALS cases (Supplementary Figure 1, and Supplementary Table 1). Immunostaining, using both the 24–39Ra and the 57–72Ra antibodies, revealed diffuse misSOD1 accumulation as well as misSOD1-positive deposits in the cytoplasm of motor neurons in almost all of the SALS cases studied (Supplementary Figures 2,3, and Supplementary Table 1). Axonal, perivacuolar and nuclear misSOD1 positive immunostainings were also observed in the vast majority of SALS spinal cord sections (Supplementary Table 1). MisSOD1 species in the cytoplasm of motor neurons were also detected in SALS patients, using both C4F6 and 131–153Ra antibodies, targeting the C-terminal part of the SOD1 protein (Supplementary Figures 4, 5, and Supplementary Table 1). MisSOD1-positive immunostaining was also frequently observed in large myelinated fibers in the lumbar ventral horn for the vast majority of SALS patients using all of the tested antibodies (Fig. 3 and Supplementary Table 1).

**Figure 4:**
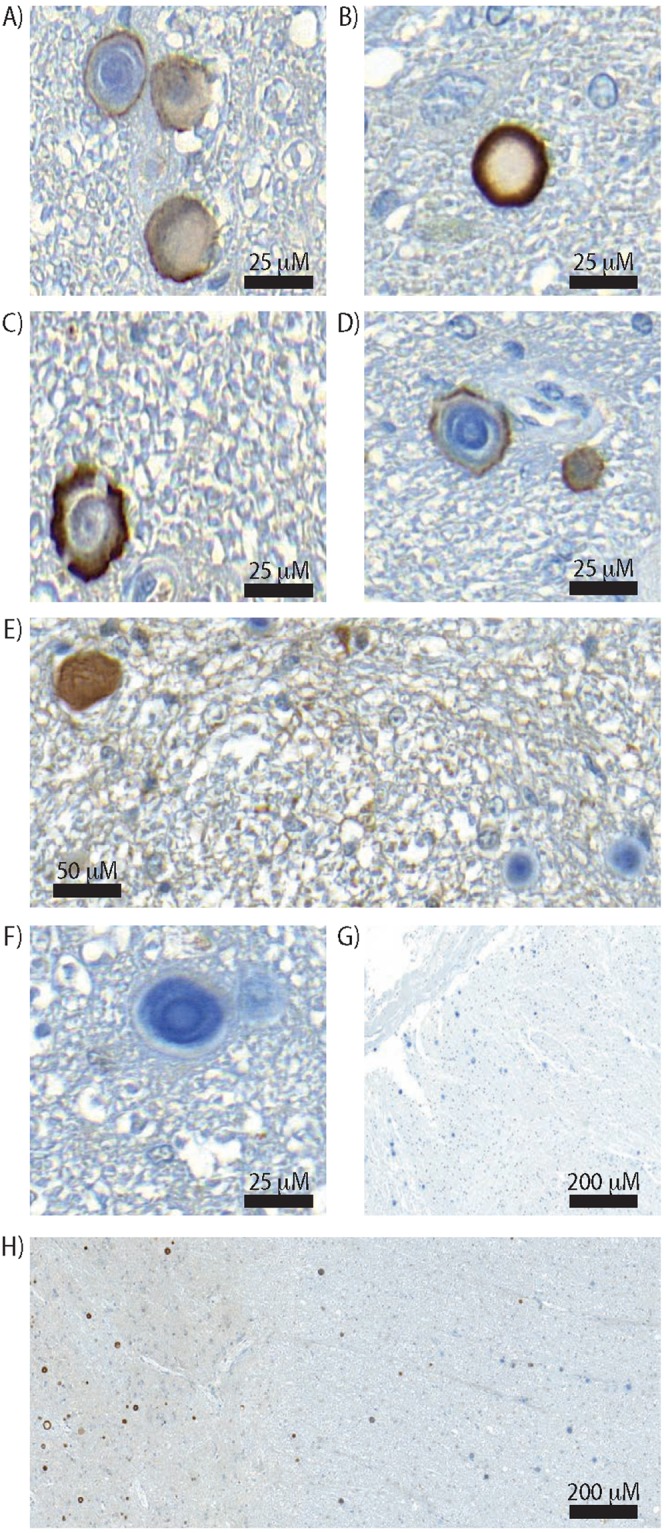
Detection of misSOD1-positive ring-like structure in the SALS and control individuals Representative images of misSOD1-positive ring-like structures in the extracellular space. (**A**–**D**) misSOD1-positive serpiginous structures detected in SALS cases detected in spinal horn grey matter. (**E**–**G**) misSOD1-negative CA-like structures detected in SALS cases located in the spinal cord tissue section periphery. (**H**) Spinal ventral horn grey matter accumulation of misSOD1-positive CA-like structures and, peripheral white matter negative misSOD1 CA-like deposits within the spinal cord.

Interestingly, misSOD1-positive ring-like immunostainings, surrounding corpora amylacea (CA)-like structures, were densely located within the grey matter of spinal anterior horns, whereas misSOD1-negative CA-like structures were rather observed at the spinal cord periphery (Fig. 4).

**Figure 5:**
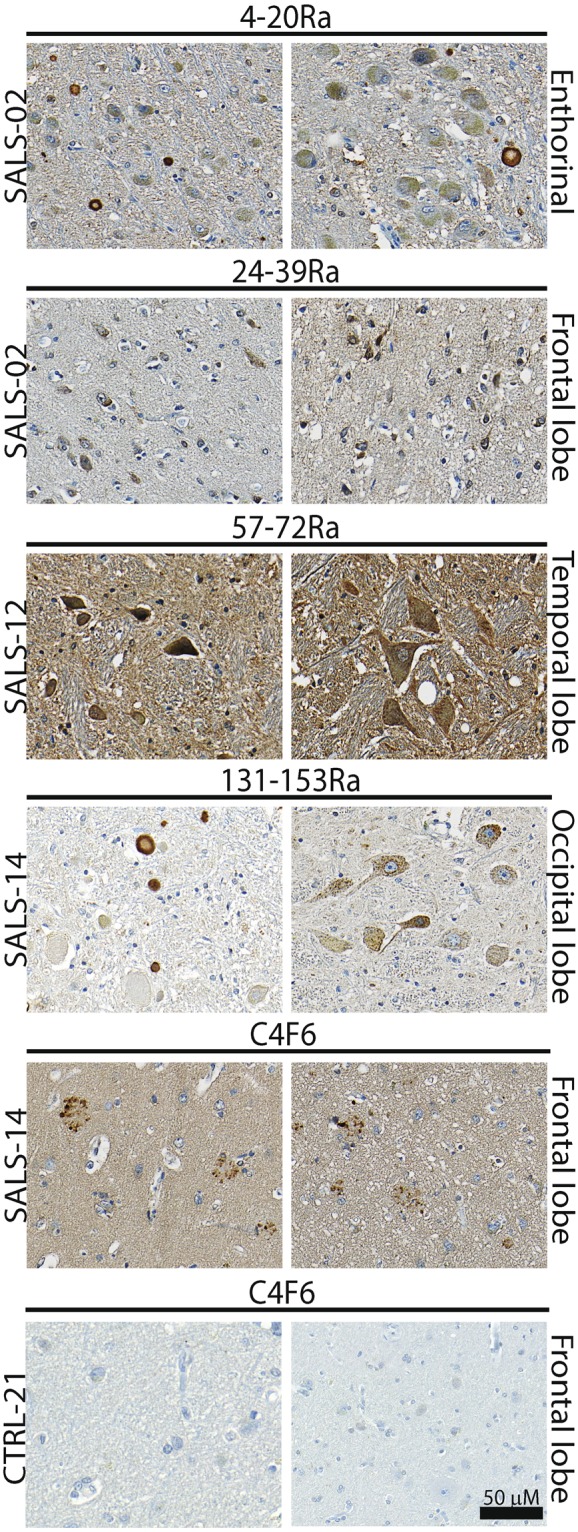
Immunodetection of misfolded SOD1 aggregates detected in brain tissue sections of SALS patients by immunohistochimestry using the misfolded SOD1/conformational-specific 4–20Ra, 24–39Ra, 57–72Ra, C4F6 and 131–153Ra antibodies Brain tissue sections from SALS patients and non-ALS control individuals immunostained using 5 different misSOD1/conformational-specific antibodies. Two representative pictures per SALS patients and control are showed. Different brain areas were analysed and misSOD1-positive accumulation can be observed.

MisSOD1 accumulation was also detected with at least 2 or more of the above-described patterns of staining, for all of the tested conformation specific misSOD1 antibodies, in post-mortem brain tissue sections (frontal, temporal and occipital lobes as well as enthorinal sections) from SALS patients (Fig. 5). MisSOD1 immunoreactivity was negligable in the 2 individuals with Alzheimer’s disease and in the non-neurological controls. Furthermore, no misSOD1-positive staining of CA-like structures was observed in control material.

### Immunocapture of misSOD1 from SALS spinal cord

**Figure 6:**
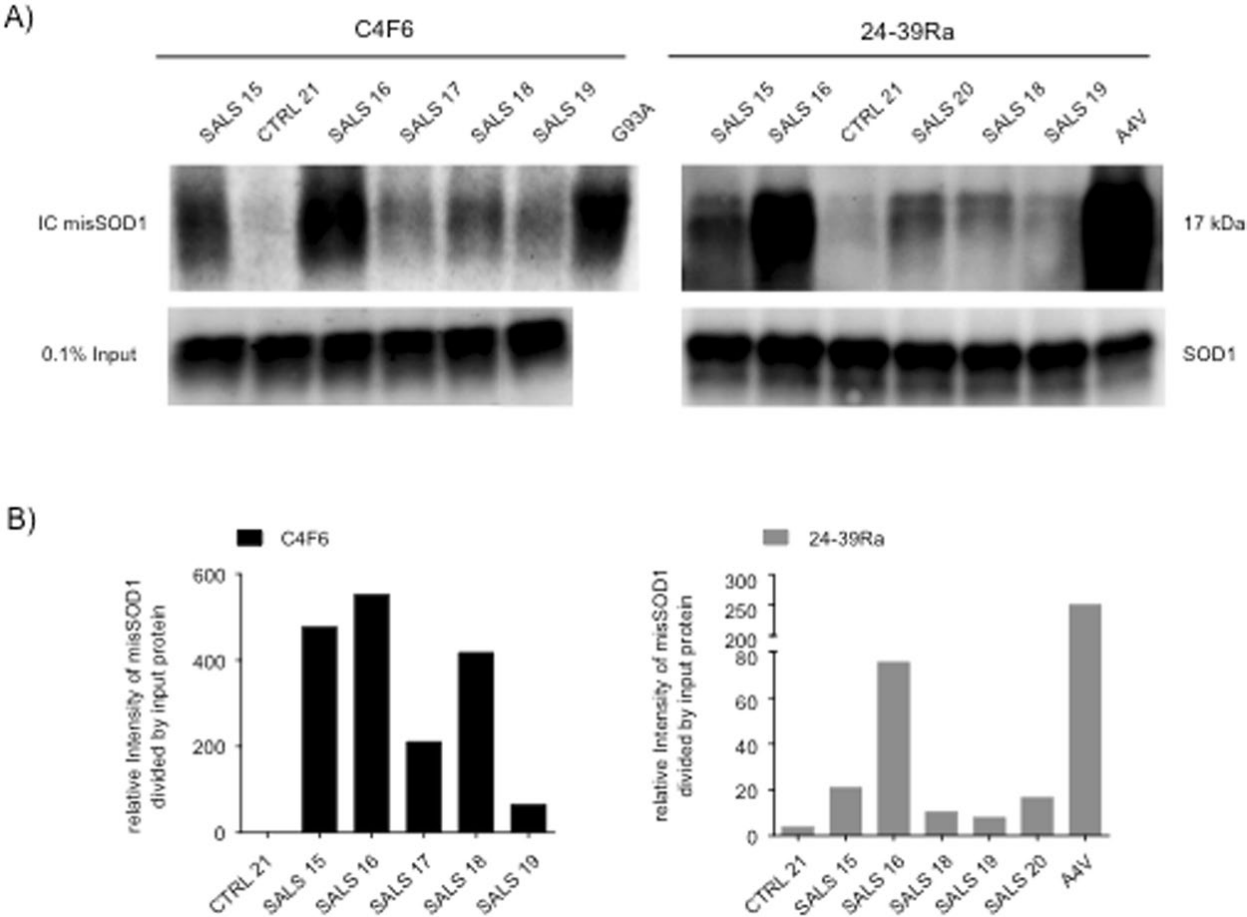
Immunocapture of misSOD1 in SALS spinal cord tissue samples using the C4F6 and 24–39Ra misSDO1 specific antibodies (**A**) Immunocapture of misSOD1 in total spinal cord protein extracts, obtained from SALS patients, A4V-*SOD1* FALS patient, non-ALS control individual and transgenic mice over-expressing G93ASOD1 mutant protein is illustrated. (**B**) Quantification data of the immunocaptured misSOD1 protein shown in panel A. Immunodetection of the transferred immunucaptured products, using the 24–39Ra and the C4F6 misSOD1/conformational specific antibodies, revealed that both antibodies yielded clear and positive immunoreactivity signals and a high degree of specificity whilst only very low levels were detected in spinal cord extracts collected from the non-ALS control individual. A fraction of the total amount of proteins used (0.1%) was also run on a SDS–PAGE to validate that an equal amount of protein was loaded from each sample. Note that, due to the availability of patient materials, SALS17 and SALS20 spinal cord extracts were only immunocaptured using either the C4F6 or 24–39Ra antibodies.

To validate the presence of misSOD1 in SALS spinal cord protein extracts, we used an independent method to immunocapture misSOD1 from whole tissue extracts with the C4F6 and 24–39Ra antibodies. Although all misfolded/conformational SOD1 antibodies used in this study worked in immunohistochemistry, the C4F6 and 24–39Ra misfolded/conformational SOD1 antibodies were the best working antibodies for this specific experiment. Available frozen and unfixed spinal cords from SALS patients were used. As positive controls we also included spinal cord tissue from an A4V *SOD1* mutation carrier and from an end-stage G93A*SOD1* mouse. Spinal cord protein extract, collected from one non-neurological control, was used as a negative control. At a similar level of SOD1 in the input sample, significant amounts of misSOD1 were immunocaptured from SALS and A4V *SOD1* spinal cords (Fig. 6). However, no misSOD1 was detected above background threshold in the non-neurological control lysate.

## Discussion

To ascertain the potential importance of misfolded SOD1 in the pathology in SALS, we conducted blinded, controlled, histopathological and biochemical analyses of misSOD1 in brain and spinal cord samples from a FALS A4V-*SOD1* patient and 19 SALS patients (13 analyzed by IHC and 6 by IC/Western blotting). Non-neurological control as well as non-ALS neurological control individuals showed no specific misSOD1 positive immunostaining patterns (Supplementary Table 1). In SALS individuals, variability in misSOD1 staining patterns was observed, which were found to be highly specific to SALS, whereas no misSOD1 immunoreactivity was detected in all tested control individuals including neurological Alzheimer patients. Detection of misSOD1 in available SALS spinal cords was also confirmed by immunocapture analysis. Hence, differential staining patterns may correspond either to a pathological – clinical correlation, or reflect the presence of different misSOD1 species.

An evolving hypothesis has proposed a common final neurodegenerative pathway shared by at least some FALS and SALS cases that involves cytotoxic, non-native conformers of SOD1. This is supported by several studies, which have demonstrated that: (1) wtSOD1 acquires properties of ALS-linked mutant human SOD1 species^27,32^; (2) Mice that overexpress wild-type human SOD1 at high rate develop both SOD1 aggregation and a fatal ALS-like disease^24^; (3) Expression of human wtSOD1 exacerbates disease in transgenic mice expressing different mutant SOD1s^34,35^; (4) Aberrant cytoplasmic TDP-43 or FUS accumulation can trigger misfolding of the human wtSOD1 protein^8^; and (5) Misfolded forms of human wtSOD1 have been detected in post-mortem CNS tissue of both SALS and FALS without *SOD1* mutations^8,19,26,28^. However, the presence of misSOD1 in SALS is disputed (Table 3). Variability in the fixation and storage of autopsy material and in the standardization of immunostaining protocols may be one of the reasons for such divergent interpretations. Regardless, the difficulty to detect misSOD1 species in SALS or the presence of misSOD1 immunostaining in control individuals does not formally rule out its role as a pathological component in SALS. Lack of compliance with the published instructions for the staining of misSOD1 aggregates and with the actual proposed standardized immunodetection guidelines (Supplementary Table 2), which may lead to false-negative or false-positive results, are most likely the reasons for the divergent interpretations.

A common denominator between all published studies evaluating the presence of misSOD1 species in SALS is the lack of standardization in IHC experimental protocols. This may lead to inconsistent results and so far standardized protocols for the evaluation of SOD1 staining patterns has been limited by a lack of standard criteria defining misSOD1 immunostaining. This has made it difficult to compare results of retrospective or prospective studies. To overcome this drawback, we have sought to establish clear experimental methods and guidelines for the comparison of misfolded SOD1- and conformational-specific antibodies (summarized in Table 4 and fully described in the Supplementary Table 2). We suggest that the adherence to these guidelines will aid in the interpretation of staining patterns with misSOD1/conformational antibodies and propose that the same guidelines might also be applicable to other misfolded-specific and conformational SOD1 antibodies.

In contrast to our results, Da Cruz and colleagues firmly excludes the possibility that misfolded SOD1 is a common feature of sporadic ALS^42^. The authors found no difference between SALS and controls by IHC, and an absence of misSOD1 immunoreactive signal by IF in any of the tested cases. It is important to highlight here that this discrepancy with our actual data depends on differences in the methodology used for both IHC and IF protocols. As a matter of fact, Da Cruz and colleagues used Tris/EDTA based-buffers to retrieve antigens, which most likely led to false-positive misSOD1 staining in their controls. Indeed, EDTA is known to be a chelator of bivalent metals that may interfere with the proper folding of metalloproteins such as SOD1, and may therefore explain the presence of false-positive misSOD1 signals^20,43–45^. The use of the reducing agent DTT, preventing intra-molecular disulfide bond formation necessary for the proper folding of the protein, could also explain the false-positive detection of misSOD1 by immunocapture obtained by Da Cruz and colleagues in non-ALS controls. Moreover, the antigen retrieval step, shown to be crucial when working with misSOD1 antibodies, was omitted during the IF protocol used by Da Cruz and colleagues, as well as questionable antibody working dilutions (e.g. 1/60,000) for the detection of misSOD1, could also explain the lack of misSOD1 immunoreactive signal.

Different patterns of misSOD1 immunostaining were observed in this study, including nuclear misSOD1 accumulation and perivacuolar misSOD1-positive ring-like structures in the extracellular space (Figs 3 and 4). Although nuclear wtSOD1 as well as misSOD1 nuclear expression as been previously reported^28,46^, clear evidence of a nuclear function of SOD1 protein, either native or misfolded, has not been ascertained. Taking into account the serpiginous aspect and the size of the detected misSOD1 positive ring-like structures, it is likely that these could be corpora amylacea (CA), which are small hyaline masses of unknown origin. CA are round extracellular structures of 10 to 50 μm in diameter and are frequently found beneath the pia matter within the normal aging brain as well as in a variety of neurological conditions including AD, multiple sclerosis, hippocampal sclerosis and epilepsy^47–53^. Their composition includes ubiquitin, heat-shock proteins, myelin basic protein, NeuN, S100 proteins, alpha-synuclein (for review see^54^) which suggests protein contents probably originates from neuron and/or neuropil degradation products. Their ability to calcify over time is an additional argument in favour of this hypothesis. CA were shown to be less abundant in ALS or in Parkinson patients as compared to CNS samples from AD patients^51^. In contrast, misSOD1-positive CA-like structures were frequently observed in spinal cord CNS samples from SALS patients in the present study (Supplementary Table 1). On the other hand, we were not able to detect these misSOD1-positive structures in the neurological and non-neurological controls, indicating that the detected misSOD1 immunoreactive signals may be specific to SALS. They can be witnesses of the evolution of the disease but can also have an alternative protective function by sequestering aggregated proteins. The precise role or relevance of misSOD1-positive CA-like structures in our study remain elusive. Interestingly, misSOD1 CA-like structures were also densely located within the grey matter of spinal anterior horns in SALS patients while negative CA were often located at the spinal cord periphery (Fig. 4). Our data support the idea that different CA counterparts, arising from different origin, may exist^55^ (Fig. 4 and Supplementary Figure 6). This study interestingly showed that CA lying beneath the pia matter, often larger in size, are unaffected by changes in the neuronal population, whereas CA lying in the grey matter may be more responsive to neuronal loss. To date, our data are in favour of their specific character in SALS and encourages to consider them as positive marker of the disease (pattern 4 of the reading grid) even if further studies are necessary in order to clearly delineate their role and their specificity in ALS.

## Material and Methods

### Animals

Transgenic mice overexpressing G93A mutant *SOD1* (www.jax.org/strain/002726) were maintained and cared for according to the guidelines of the Animal Care and Use Committee of Umeå University, Sweden. The Regional Ethical Committee for Animal Experiments approved experimental protocols involving animals beforehand, under a project with reference A49–14.

### Human samples and neuropathological examination

This study was performed in accordance with the Declaration of Helsinki (WMA, 1964) and approved by the appropriate national ethical review boards in Canada (Ethical research board of the CHU de Québec. Protocol number: 2012-1316. For more information, please contact gurecherche@chuq.qc.ca) or Sweden (Ethical Review Board Ref. No. 14-17-31 M). Human autopsies were performed as described^56^. Informed consent for study participation has been obtained from each participant.

CNS tissue was obtained from autopsy material from 19 SALS and one A4V-*SOD1* FALS patient, as well as 2 non-neurological control individuals and 2 neurological (Alzheimer’s Disease) controls (see Table 1 for clinical information). The ALS patients met the El Escorial criteria for clinically definite, probable or laboratory supported ALS^57^. All died from respiratory failure, some with concomitant pneumonia. None had received treatment with invasive ventilation through a tracheotomy, or experimental therapy apart from treatment with riluzole. A diagnosis of FALS or SALS was deduced after collecting genealogical and medical data from at least three prior generations in accordance with Byrne *et al*.^58^. All ALS patients were also screened for mutations in a panel of known ALS-causing genes including *SOD1*, *TARDBP*, *FUS* and a hexanucleotide-repeat expansion in *C9ORF72* (for details see^59–63^). Analyses were performed on genomic DNA extracted from peripheral blood leucocytes. Post-mortem tissue samples from one non-neurological individual and from 2 Alzheimer’s disease patients were obtained from the NIH Neurobiobank at the University of Maryland, Baltimore, MD. Post-mortem CNS tissues were fixed by immersion in 20% buffered formalin, or stored unfixed in a −80 °C freezer until use. Paraffin-embedded material was used for immunohistochemical analyses. Sectioning of SALS and control post-mortem tissues (10 mm thickness) was performed at the CHU de Quebec. All slides were encoded and sent to Umea University for immunohistochemical analyses.

### Immunohistochemistry

Sections were stained using the automated BenchMark ULTRA (Ventana Tucson, AZ, USA). Deparaffinization of tissue sections was performed by heating at 72 °C for 12 minutes in Ventana EZ solution. Antigen retrieval was done using a sodium citrate solution (CC2, Ventana Tucson, AZ, USA) at 91 °C for 44 minutes (24 minutes for 57–72Ra antibody) to obtain the optimal signal to noise ratio. Primary antibodies were incubated for 32 minutes at 37 °C. Sections were then stained using the *ultraView* Universal DAB detection kit and counterstained with hematoxylin and lithium carbonate (Bluing reagent). Slides were dehydrated with alcohol and xylene before mounting. Imaging was performed using a digital slide scanner. Antibodies, dilutions and antigen retrieval incubation times are listed in Table 2.

### Immunocapture

End-stage mutant G93A*SOD1* transgenic mice were sacrificed by intraperitoneal injection of pentobarbital. The spinal cord was isolated by flushing with saline and homogenized immediately in 25 volumes of ice-cold immunocapture (IC) buffer (PBS, 40 mM iodoacetamide (IAM, Thermo Scientific, Rockford, IL, USA), Complete EDTA-Free Protease Inhibitor Cocktail (Roche Diagnostics, Mannheim, Germany) and 0.5% Nonidet P-40 using a tissue homogenizer followed by sonication. Lysates were centrifuged at 25,000 g for 30 min. at 4 °C and supernatants were stored at −80 °C until analysis.

Segments of un-fixed frozen lumbar spinal cord from patients were used for immunocapture. Weighed spinal cord sections were homogenized in 10 volumes of IC buffer as described above, centrifuged at 1,000 g for 10 min. at 4 °C and the resulting supernatant was analyzed by immunocapture.

Anti-human SOD1 antibodies (24–39Ra or C4F6) were cross-linked to Dynabeads M-270 Epoxy with the Dynabeads Antibody Coupling Kit (Invitrogen, California, USA). Beads were isolated with a magnet, washed to remove unbound antibodies and equilibrated with IC buffer. Antibody-coated beads were incubated with equal volumes of spinal cord extracts for 1 hour at 23 °C. The beads were washed 5 times with IC buffer and samples were eluted by boiling in 1x sample buffer containing 40 mM IAM. Immunocaptured proteins and the spinal cord extracts were analyzed using non-reduced SDS-PAGE and western blotting as previously described^64–66^.

The different binding regions, recognized by the misfolded/conformational SOD1 antibodies were visualized using the SOD1 1AZV protein data bank entry^67^ with the Chimera protein structure visualization system^68^. Note that the minimal binding epitope for C4F6 contains, but not necessarily restricted to, amino acids 90–93^41^.

## Electronic supplementary material

Supplementary information

## Data Availability

All data generated or analysed during this study are included in this published article.
