# Supplementary material for: Misfolded SOD1 pathology in sporadic Amyotrophic Lateral Sclerosis
Source: Sci Rep. 2018 Sep 21;8:14223. doi: 10.1038/s41598-018-31773-z (PMC6155098; doi:10.1038/s41598-018-31773-z)
Supplement: Supplementary file 1 — Supplementary information [file 41598_2018_31773_MOESM1_ESM.pdf]

Supplementary information

Misfolded SOD1 pathology in sporadic Amyotrophic Lateral Sclerosis

Bastien Paré[1,2], Manuela Lehmann[3], Marie Beaudin[4], Ulrika Nordström[3], Stephan Saikali[5], Jean-Pierre Julien[6,7], Jonathan D. Gilthorpe[3], Stefan L. Marklund[8], Neil R Cashman[9], Peter M. Andersen[3], Karin Forsberg[10], Nicolas Dupré[4], Peter Gould[5], Thomas Brännström[10] and François Gros-Louis [1, 2, *]

1. Laval University Experimental Organogenesis Research Center / LOEX, Division of Regenerative Medicine, CHU de Québec Research Center – Enfant-Jésus Hospital, Québec, Canada
2. Department of Surgery, Faculty of Medicine, Laval University, Québec, Canada
3. Department of Pharmacology and Clinical Neuroscience, Umeå University, Umeå, Sweden
4. Neuroscience Division of the CHU de Québec and Department of Medicine of the Faculty of Medicine, Laval University, Québec, QC, Canada
5. Department of Medical Biology, Division of Anatomic Pathology and Neuropathology, CHU de Québec, Hôpital de l'Enfant-Jésus, Québec, Canada
6. Department of Psychiatry and Neuroscience, Laval University, Québec City, Québec, Canada
7. Centre de Recherche CERVO, Québec City, Québec, Canada
8. Department of Medical Biosciences, Clinical Chemistry, Umeå University, Umeå, Sweden
9. Department of Medicine (Neurology), Brain Research Center, University of British Columbia, Vancouver, BC, Canada
10. Department of Medical Biosciences, Pathology, Umeå University, Umeå, Sweden

* Corresponding author:
Francois Gros-Louis, PhD
Centre de Recherche du CHU de Québec – Hôpital de l'Enfant-Jésus (LOEX)
1401, 18e rue
Québec, Canada, G1J 1Z4
Francois.gros-louis@fmed.ulaval.ca

Supplementary Table 1: Summary of misSOD1 accumulation in human patient spinal cords by following standardized immunohistoschemistry

	4-20RA	24-39RA	57-72RA	C4F6	131-153RA
**FALS-SOD1A4V**	(1,3,5)	(1, 2, 4)	(1, 2, 5)	(1, 2, 4)	(1, 2, 5)
**SALS02**	(4, 5)	(1, 2, 4, 5)	ND	(1, 2, 3)	ND
**SALS03**	ND	(1, 2, 4, 5)	(3, 4, 5)	(1, 2, 3)	(1, 3, 4)
**SALS04**	ND	(1, 4, 5)	ND	(1, 5)	(1, 3, 4, 5)
**SALS05**	ND	(2, 5)	(3, 4, 5)	ND	(1, 4)
**SALS06**	(1, 3, 4, 5)	(1, 4, 5)	(1, 3, 4, 5)	(3)	(1, 3, 4, 5)
**SALS07**	(1, 4, 5)	(4, 5)	(3, 4, 5)	(1, 3, 5)	(2, 3, 4, 5)
**SALS08**	(4, 5)	(4, 5)	ND	(4)	(4, 5)
**SALS09**	(3, 4, 5)	(3, 4, 5)	(3, 4, 5)	ND	(3, 4, 5)
**SALS10**	(0)	(1, 2, 3, 4, 5)	(3, 4, 5)	(3)	(1, 2, 3, 4, 5)
**SALS11**	(4)	(1, 4)	(4, 5)	ND	(1, 4, 5)
**SALS12**	(0)	(2, 3, 5)	ND	(1,5)	ND
**SALS13**	(0)	(1, 5)	ND	(1, 4, 5)	(1, 4, 5)
**SALS14**	ND	ND	(1, 5)	(1, 3, 4)	(1, 3, 5)
**CTRL21**	(0)	(0)	ND	ND	(0)
**CTRL22**	(0)	ND	ND	(0)	(0)
**CTRL23**	(0)	(0)	(0)	(0)	ND
**CTRL24**	(0)	(0)	(0)	ND	ND

0) no detected immunoreactive signal, 1) misSOD1 cytoplasmic accumulation, 2) cytoplasmic misSOD1-positive deposits, 3) axonal misSOD1-positive accumulation, 4) ring-like misSOD1-positive accumulation, and 5) misSOD1-positive nuclear accumulation. ND: not determined

Supplementary Table 2: Detailed description of the proposed guidelines

	Guidelines	Complete description
1	To use more than one misSOD1 specific antibody before concluding any results	As not all conformational specific misSOD1 antibodies can equally detect different misfolded SOD1 species of WT SOD1, which can be attributed to antibody affinity or antibody mapping to different epitopes, using more than one misSOD1 antibody must be considered
2	To test and use optimal antibody working concentrations	Antibody affinity can be affected by many variables including temperature, pH and buffer constituents. Therefore, the optimal working concentration of each individual antibody must be determined for experimental conditions. Even though some commercial antibodies have recommended dilutions for various applications, optimization is often required to reduce signal to noise ratio and obtain the best staining with minimal background
3	To optimize antigen retrieval time for each antibody [1]	Antigen retrieval time is a crucial aspect in immunohistochemistry, as antigen may often by masked by a variety of chemical modifications, following tissue fixation and embedding, that can reduce the detectability of proteins. As protocols of cell sample preparation and fixation, used in cytopathology, are not standardized across laboratories, we recommend to always optimize the antigen retrieval procedure to enhance antigenicity for both IHC and immunofluorescence. Such optimizations will necessarily means the addition of positive and negative controls
4	To always use citrate-based instead of TRIS/EDTA-based buffers	Considering the denaturing effect of certain antigen retrieval buffer on the SOD1 native protein, the use of appropriate citrate-based buffer solution must be considered. For instance, EDTA-based buffer is to be proscribed as it is known to be a metal ions scavenger that may interfere with the proper folding of metalloprotein such as SOD1, and give raise to false positive signals [2]. Reducing agents such as dithiothreitol (DTT) and β-mercaptoethanol, known to alter the folding and precipitation capacity of the wild-type protein [3], must also be proscribed when studying misSOD1 regardless of the detection technique used [2,4–6]
5	To test different central nervous system regions (cervical, lumbar, thoracic spinal cord sections and other brain regions)	As site of onset and severity of the disease can be highly heterogenous from patient to patient, we recommend to always perform histopathological examination on different CNS regions including brain (as the motor cortex and frontal lobe) and spinal cord sections. Examination of spinal cord must also include at least cervical, lumbar and thoracic sections
6	To perform, in parallel, Hematoxylin/Eosin coloration on adjacent sections, as well as IHC control with no primary antibody	Performing unspecific HE coloration and control IHC without primary antibody will help in the interpretation of results confirming that the detected misSOD1-positive aggregates are not unspecific chromophilic structures

1. Ayers JI, Xu G, Pletnikova O, Troncoso JC, Hart PJ, Borchelt DR. Conformational specificity of the C4F6 SOD1 antibody; low frequency of reactivity in sporadic ALS cases. Acta Neuropathol Commun [Internet]. 2014;2(1):55. Available from: http://www.actaneurocomms.org/content/2/1/55
2. Gros-Louis F, Soucy G, Larivière R, Julien JP. Intracerebroventricular infusion of monoclonal antibody or its derived Fab fragment against misfolded forms of SOD1 mutant delays mortality in a mouse model of ALS. J Neurochem. 2010;113(5):1188–99.
3. Furukawa Y, Kaneko K, Yamanaka K, Nukina N. Complete Loss of Post-translational Modifications Triggers Fibrillar Aggregation of SOD1 in the Familial Form of Amyotrophic Lateral Sclerosis. J Biol Chem. 2008;283(35):24167–76.
4. Strange RW, Yong CW, Smith W, Hasnain SS. Molecular dynamics using atomic-resolution structure reveal structural fluctuations that may lead to polymerization of human Cu-Zn superoxide dismutase. Proc Natl Acad Sci U S A [Internet]. 2007;104(24):10040–4. Available from: http://www.pnas.org/content/104/24/10040.full
5. Strange RW, Antonyuk S, Hough MA, Doucette PA, Rodriguez JA, Hart PJ, et al. The structure of holo and metal-deficient wild-type human Cu, Zn superoxide dismutase and its relevance to familial amyotrophic lateral sclerosis. J Mol Biol. 2003;328(4):877–91.
6. Johansson A-S, Vestling M, Zetterström P, Lang L, Leinartaitė L, Karlström M, et al. Cytotoxicity of superoxide dismutase 1 in cultured cells is linked to Zn2+ chelation. PLoS One [Internet]. 2012;7(4):e36104. Available from: http://www.pubmedcentral.nih.gov/articlerender.fcgi?artid=3338499&tool=pmcentrez&rendertype=abstract

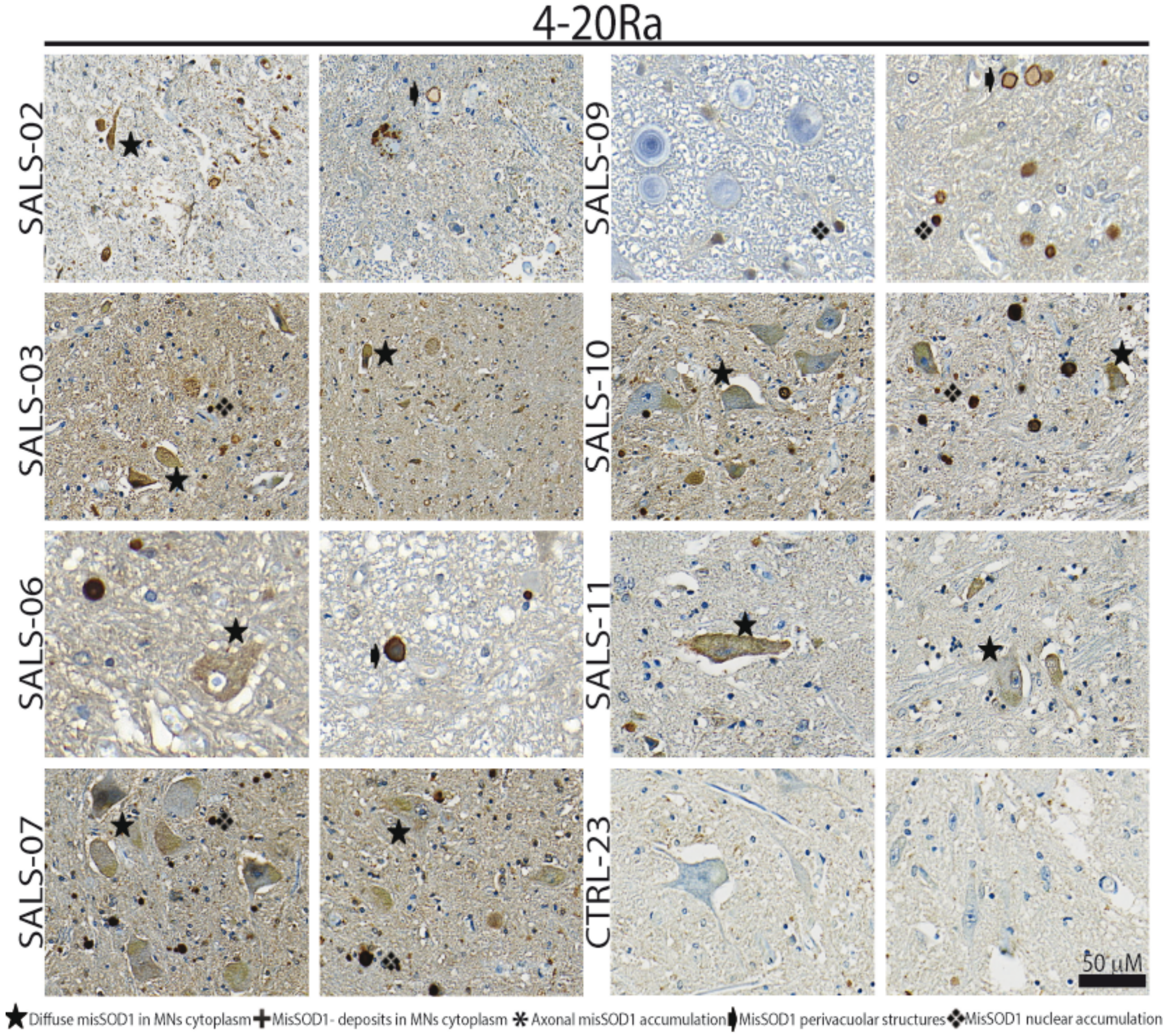

Supplementary Figure 1: Immunodetection of misfolded SOD1 species detected in post-mortem tissue sections from SALS patients by immunohistochimestry using the misfolded SOD1-specific 4-20Ra antibody

Spinal cord sections from SALS patients and non-ALS control individual immunostained using the 4-20 rabbit polyclonal misSOD1-specific antibody. Two representative pictures for each SALS patients and control are showed. Representative misSOD1 staining patterns are highlighted in each display panels and are labeled as followed: ★ diffuse misSOD1 staining in the cytoplasm of motor neurons; + misSOD1-positive deposits in the cytoplasm of motor neurons; * axonal misSOD1 accumulation; ➡ misSOD1-positive perivacuolar ring-like structures; ❖ shows misSOD1 nuclear accumulation. Scale bar: 50 µm.

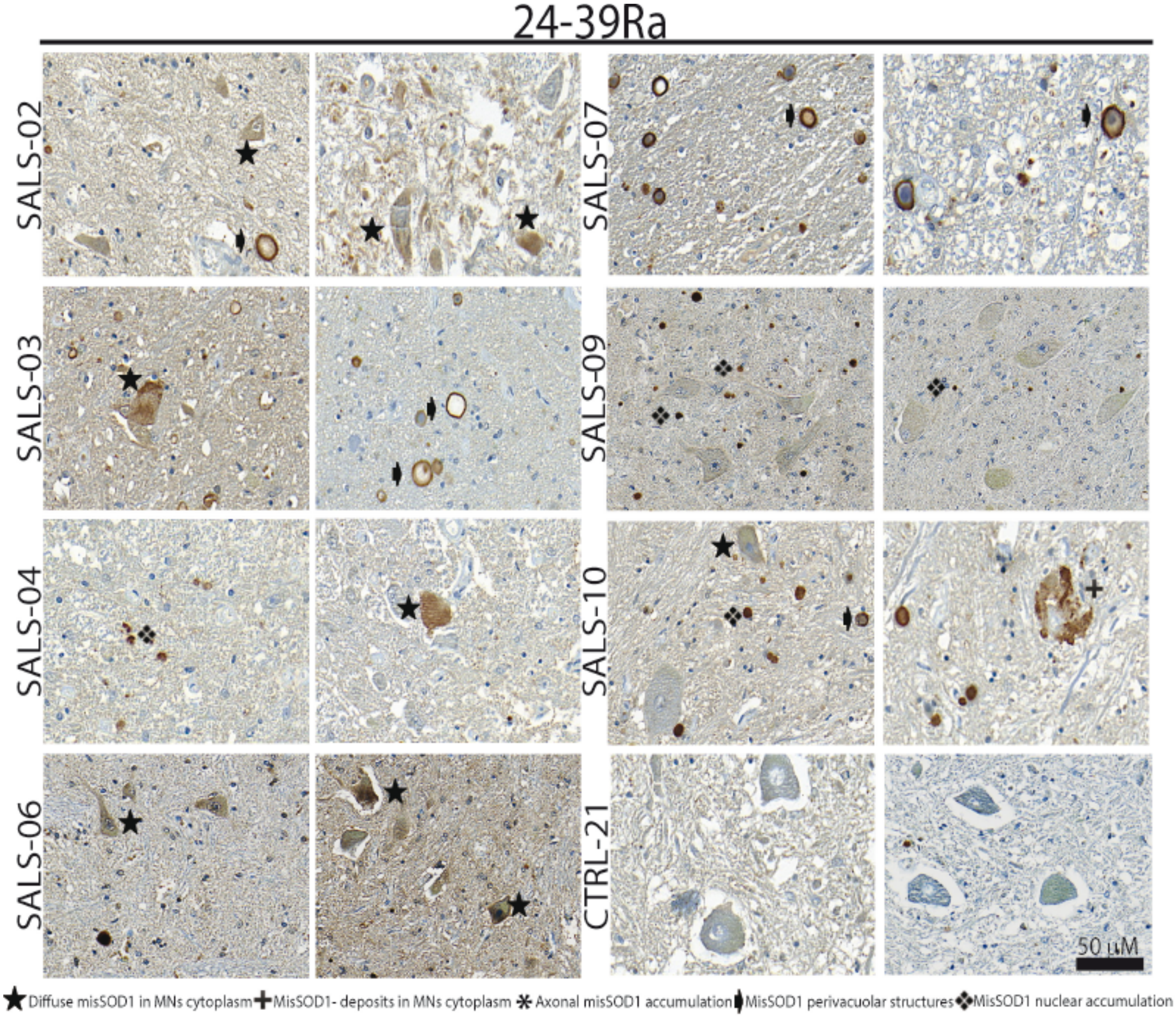

Supplementary Figure 2: Immunodetection of misfolded SOD1 species detected in post-mortem tissue sections from SALS patients by immunohistochimestry using the misfolded SOD1-specific 24-39Ra antibody

Spinal cord sections from SALS patients and non-ALS control individual immunostained using the 24-39 rabbit polyclonal misSOD1-specific antibody. Two representative pictures for each SALS patients and control are showed. Representative misSOD1 staining patterns are highlighted in each display panels and are labeled as followed: ★ diffuse misSOD1 staining in the cytoplasm of motor neurons; + misSOD1-positive deposits in the cytoplasm of motor neurons; * axonal misSOD1 accumulation; ➡ misSOD1-positive perivacuolar ring-like structures; ❖ shows misSOD1 nuclear accumulation. Scale bar: 50 μm.

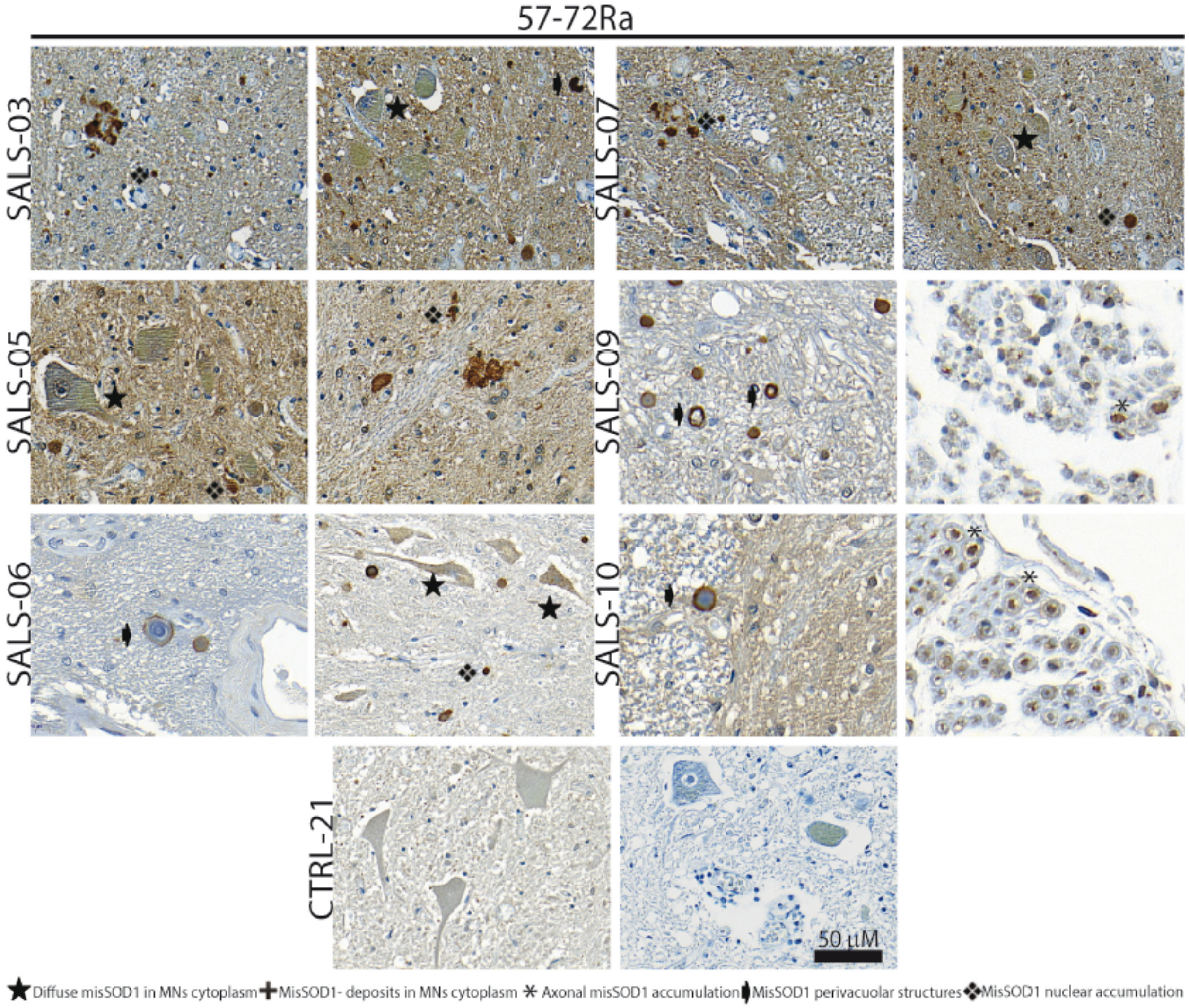

Supplementary Figure 3: Immunodetection of misfolded SOD1 species detected in post-mortem tissue sections from SALS patients by immunohistochimestry using the misfolded SOD1-specific 57-72Ra antibody

Spinal cord and ventral root sections from SALS patients and non-ALS control individuals immunostained using the 57-72 rabbit polyclonal misSOD1-specific antibody. Two representative pictures for each SALS patients and control are showed. Representative misSOD1 staining patterns are highlighted in each display panels and are labeled as followed: ★ diffuse misSOD1 staining in the cytoplasm of motor neurons; + misSOD1-positive deposits in the cytoplasm of motor neurons; * axonal misSOD1 accumulation; ➡ misSOD1-positive perivacuolar ring-like structures; ❖ shows misSOD1 nuclear accumulation. Scale bar: 50 μm.

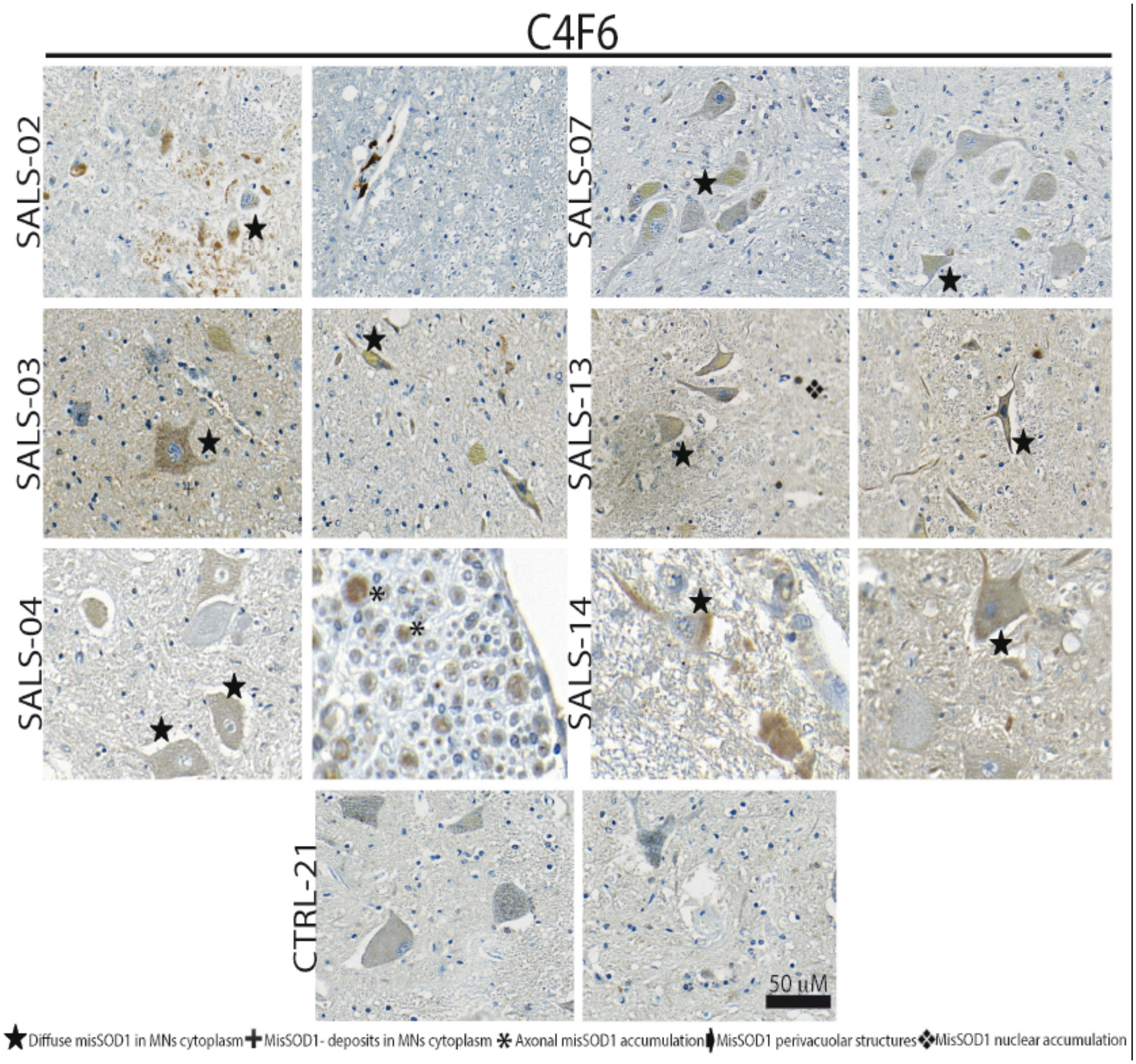

Supplementary Figure 4: Immunodetection of misfolded SOD1 species detected in post-mortem tissue sections from SALS patients by immunohistochimestry using the SOD1 conformation-specific C4F6 antibody

Spinal cord and ventral root sections from SALS patients and non-ALS control individuals immunostained using the C4F6 mouse monoclonal misSOD1-specific antibody. Two representative pictures for each SALS patients and control are showed. Representative misSOD1 staining patterns are highlighted in each display panels and are labeled as followed: ★ diffuse misSOD1 staining in the cytoplasm of motor neurons; + misSOD1-positive deposits in the cytoplasm of motor neurons; * axonal misSOD1 accumulation; ➡ misSOD1-positive perivacuolar ring-like structures; ❖ shows misSOD1 nuclear accumulation. Scale bar: 50 μm.

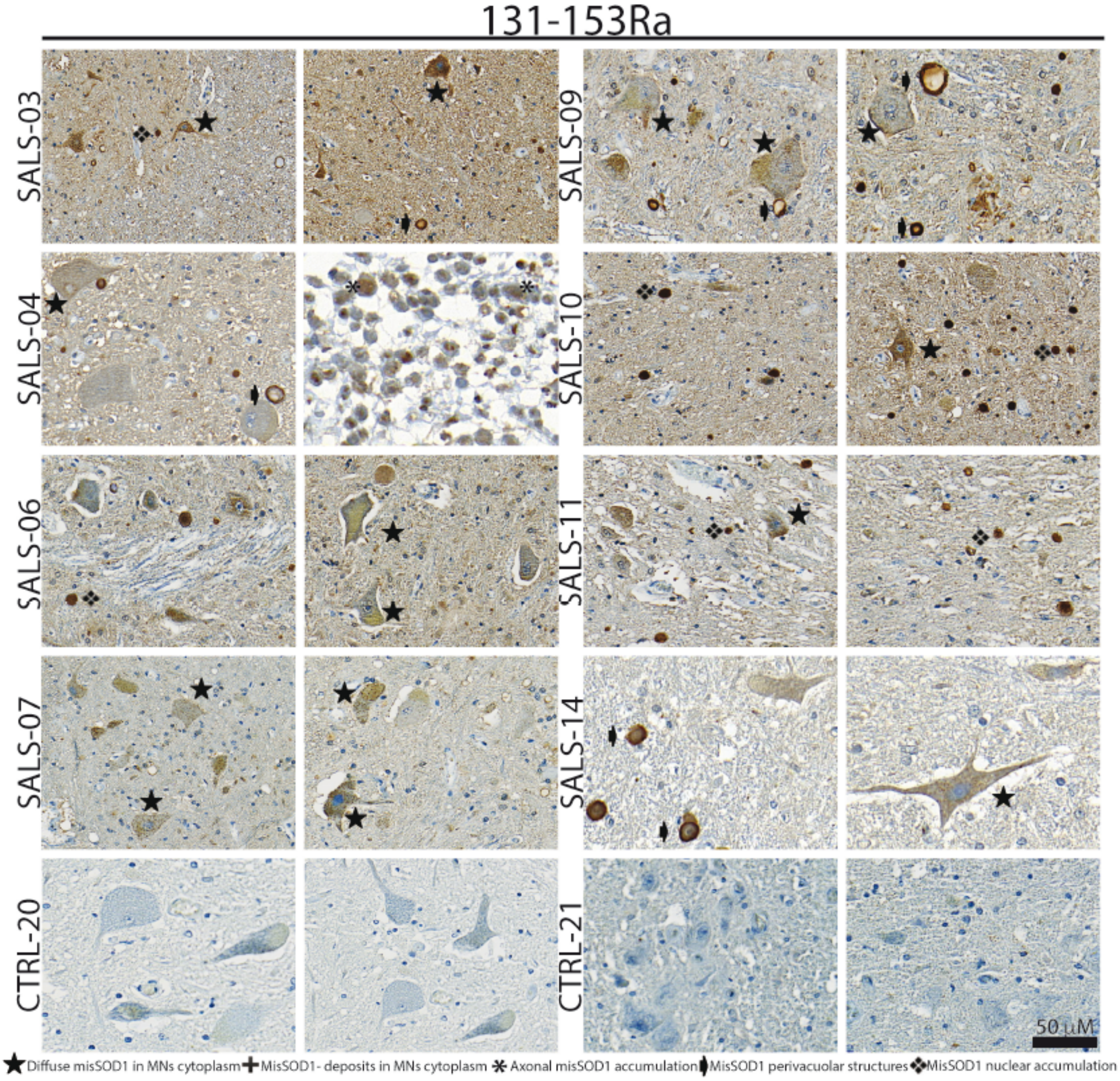

Supplementary Figure 5: Immunodetection of misfolded SOD1 species detected in post-mortem tissue sections from SALS patients by immunohistochimestry using the misfolded SOD1-specific 131-153Ra antibody

Spinal cord and ventral root sections from SALS patients and non-ALS control individuals immunostained using the 131-153 rabbit polyclonal misSOD1-specific antibody. Two representative pictures for each SALS patients and control are showed. Representative misSOD1 staining patterns are highlighted in each display panels and are labeled as followed: ★ diffuse misSOD1 staining in the cytoplasm of motor neurons; + misSOD1-positive deposits in the cytoplasm of motor neurons; * axonal misSOD1 accumulation; ➡ misSOD1-positive perivacuolar ring-like structures; ❖ shows misSOD1 nuclear accumulation. Scale bar: 50 µm.

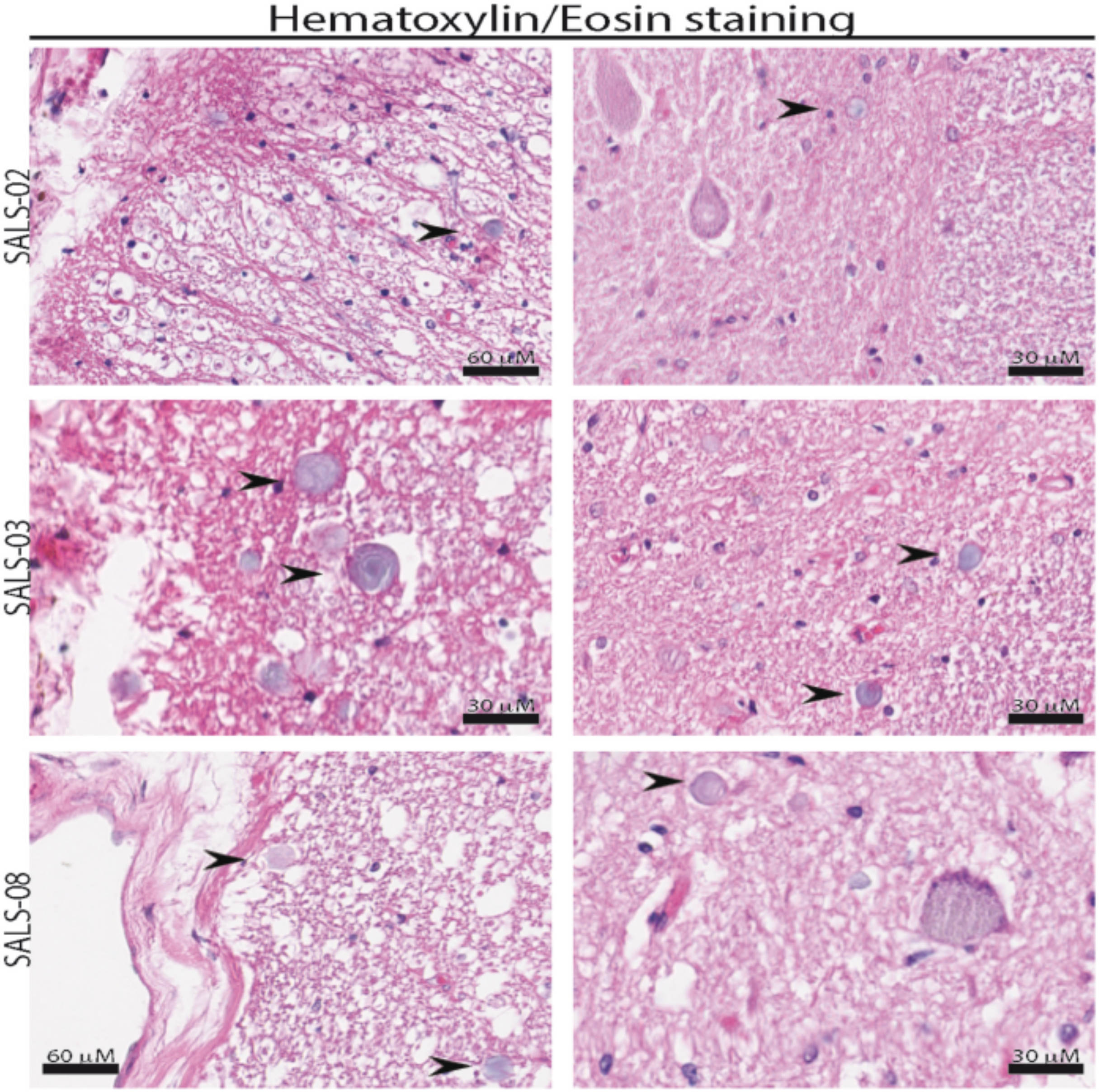

Supplementary Figure 6: Hematoxylin & Eosin stainings of corpora amylacea in sporadic ALS patients spinal cord sections

Representative hematoxylin/Eosin colorations on post-mortem spinal cord tissue sections from sporadic ALS patients. Two pictures for each patient are displayed (left panel: spinal white matter; and right panel: spinal grey matter). Corpora amylacea were mainly detected at the spinal cord periphery and beneath the pia. CA lying in the white matter and beneath the pia matter were often found to be larger in size. CA lying in the grey matter are smaller in size. Arrowheads show corpora amylacea. Scale bars are indicated in the bottom right of each displayed panel.
